# Curculio Curculis lupus: biology, behavior and morphology of immatures of the cannibal weevil *Anchylorhynchus eriospathae* G. G. Bondar, 1943

**DOI:** 10.7717/peerj.502

**Published:** 2014-07-31

**Authors:** Bruno Augusto Souza de Medeiros, Daniela de Cássia Bená, Sergio Antonio Vanin

**Affiliations:** 1 Department of Organismic & Evolutionary Biology and Museum of Comparative Zoology, Harvard University, Cambridge, MA, USA; 2 Departamento de Zoologia, Instituto de Biociências, Universidade de São Paulo, São Paulo, SP, Brazil

**Keywords:** Morphology, Larva, Weevil, Curculionidae, Palm, Seed predator, Cannibalism, Competition, Synonymy, *Butia eriospatha*

## Abstract

Weevils are one of the largest groups of living organisms, with more than 60,000 species feeding mostly on plants. With only one exception, their described larvae are typical plant-feeders, with mouthparts adapted to chewing plant material. Here we describe the second case of a weevil with early-instar larvae adapted to killing conspecifics. We have studied the life history of *Anchylorhynchus eriospathae* G. G. Bondar, 1943 (Curculioninae: Derelomini *sensu* Caldara, Franz & Oberprieler (2014)), a species whose immatures feed internally on palm flowers and fruits. We provide detailed descriptions of all immature stages, including the extremely modified first-instar larva. Unlike other weevils and later instars, this stage exhibits a flat body with very long ventropedal lobe setae, a large and prognathous head with a gula, and falciform mandibles, each with a serrate retinaculum, that are used to fight with and eventually kill other first-instar larvae. We also provide biological notes on all stages and the results of behavioral tests that showed that larval aggression occurs only among early life stages. Finally we show that adult size is highly dependent on timing of oviposition. This specialized killer first instar probably evolved independently from the one other case known in weevils, in *Revena rubiginosa* (Conoderinae: Bariditae *sensu* Prena, Colonnelli & Hespenheide (2014)). Interestingly, both lineages inhabit the same hosts, raising the possibility that both intra- and inter-specific competition shaped those phenotypes. Given the scarcity of knowledge on early larval stages of concealed insect herbivores, it is possible that our findings represent an instance of a much broader phenomenon. Our observations also allowed us to conclude that *Anchylorhynchus eriospathae* and *A. hatschbachi* G. G. Bondar, 1943 are actually the same species, which we synonymize here by considering the latter as a junior synonym (new synonymy).

## Introduction

Insect herbivores feeding on limited and contained resources such as seeds often experience strong inter and intra-specific competition. Competition might be even stronger among endophytic larvae, since they have reduced mortality caused by parasitoids and pathogens (Hawkins, Cornell & Hochberg, 1997; Cornell & Hawkins, 1995). Much attention has been devoted to the processes and outcomes of intra-specific competition in seed-feeding insects. This is usually accomplished by analyzing survival curves in controlled experiments, from which one can infer the process of competition (Smith & Lessels, 1985). However, such studies usually miss the opportunity to observe the morphology and behavior mediating the competitive interaction, and different behavioral processes could result in the same outcome (Smith & Lessels, 1985). For example, a contest outcome could be mediated by surviving larvae incidentally killing their conspecifics (e.g., Mano & Toquenaga, 2011) or by attacking them (e.g., Guedes et al., 2010; Alves-Costa & Knogge, 2005). The latter could further involve cannibalism, a common phenomenon in juvenile stages of phytophagous insects with poorly understood consequences (Richardson et al., 2010).

A spectacular example of interference competition between seed-feeding larvae is that of *Revena rubiginosa* (C. H. Boheman, 1836) (Curculionidae: Conoderinae: Bariditae *sensu* Prena, Colonnelli & Hespenheide (2014)). Adults of *Revena rubiginosa* lay their eggs inside developing fruits of the palm tree *Syagrus romanzoffiana* (Cham.) Glassman (Alves-Costa & Knogge, 2005; Guix & Ruiz, 1997). First-instar larvae have a specialized morphology, with falcate mandibles resembling those of carnivorous insects (Alves-Costa & Knogge, 2005). Even though they allegedly do not consume conspecific larvae, they actively use their mandibles to kill other individuals, and only one larva survives per fruit. Mandibles of later instar larvae have the typical form found in other Curculionidae, with strong and stout triangular mandibles used for chewing vegetable matter.

*Revena rubiginosa* is the only known case among weevils of extreme larval morphology adapted to killing conspecifics. However, this might be very common. Weevils (superfamily Curculionoidea) figure prominently among the herbivores that feed on plant reproductive organs. Within this diverse group comprising over 60,000 species (Slipiński, Leschen & Lawrence, 2011), there are numerous lineages that independently evolved this feeding habit (Oberprieler, Marvaldi & Anderson, 2007). It is likely that larvae in those taxa experience processes of intra-specific competition similar to that of *Revena*. In fact, direct interference has been observed among larvae in another seed-feeding weevil: the maize weevil *Sitophilus zeamais* (Guedes et al., 2010). Cannibalism has also been reported in other unrelated groups of weevils (namely, *Lixus* (Eluwa, 1979) and *Pissodes* (Dixon & Houseweart, 1982)). However, in those cases there is no report of cannibalism being associated with morphological specializations. Since larval morphology and behavior of the great majority of the species of weevil is unknown, we have little idea of how widespread such specializations are.

Weevils in the genus *Anchylorhynchus* C. J. Schoenherr feed internally on female flowers and developing fruits of palms in the genera *Syagrus* Mart., *Butia* Becc. (Becc.) and *Oenocarpus* Mart. (occasionally, also in the coconut *Cocos nucifera* L.) (de Medeiros & Núñez-Avellaneda, 2013; Valente & de Medeiros, 2013; Vaurie, 1954). Even though there are a few taxonomic revisions based on adults (Viana, 1975; Vaurie, 1954; Bondar, 1943; Voss, 1943) and several reports of larval feeding habits in terms of host plants (de Medeiros & Núñez-Avellaneda, 2013; Silberbauer-Gottsberger, Vanin & Gottsberger, 2013; da Silva et al., 2012; Bondar, 1943; Faust, 1894), their larvae were never described. Here we report a second case of a weevil showing morphological specializations to kill conspecifics, by studying the development, morphology and behavior of *Anchylorhynchus eriospathae* G. G. Bondar, 1943.

## Materials and Methods

### Collecting and rearing

**Figure 1:**
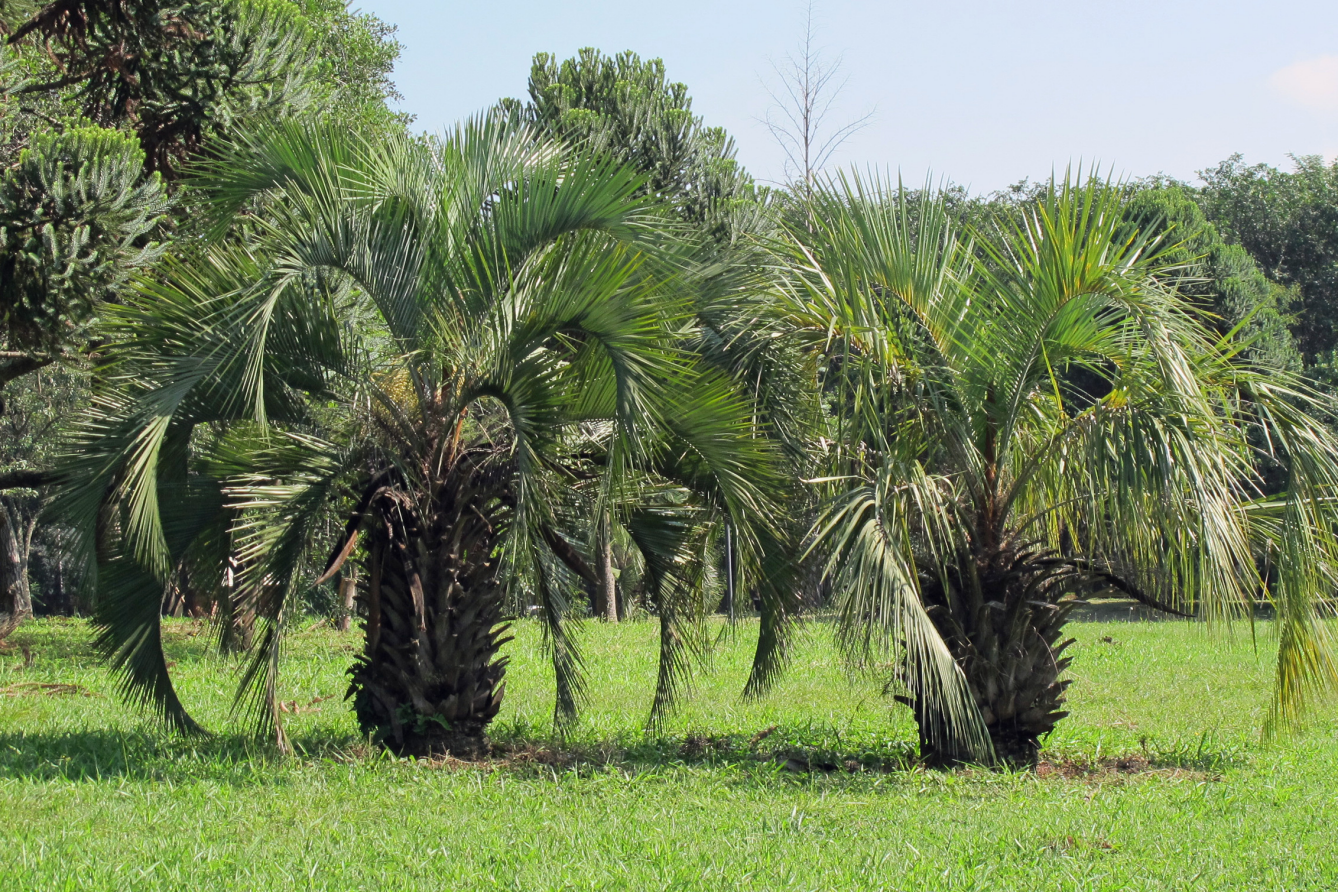
*Butia eriospatha* in the location of study. *Butia eriospatha*, host plant of *Anchylorhynchus eriospathae*, in the main campus of the Universidade de São Paulo.

We studied beetles from a population of *Butia eriospatha* (Mart. Ex Drude) Becc. found in the main campus of the University of São Paulo (Cidade Universitária Armando Salles de Oliveira). This locality is not part of the native range of this palm widely used for landscaping (Lorenzi et al., 2010), but the area known as “Praça do Relógio” in the main campus was reformulated in 1997 according to a landscape design to represent the six most important ecosystems in Brazil. One of those ecosystems is the *Araucaria* forest, and many individuals of *B. eriospatha* are planted as representatives of this ecosystem (Fig. 1). It is likely that larvae and pupae of *A. eriospathae* were introduced together with their host plants, which were transplanted as mature individuals (see results for information on pupal sites). There is a native species of palm associated with *Anchylorhynchus* in the same locality (*A. aegrotus* O. I. Fahraeus, 1843 and *A. variabilis* L. Gyllenhal, 1836 in *Syagrus romanzoffiana*), but we chose to study *B. eriospatha* because individuals start flowering while they are still short (<1.5 m high), facilitating *in situ* observations.

**Figure 2:**
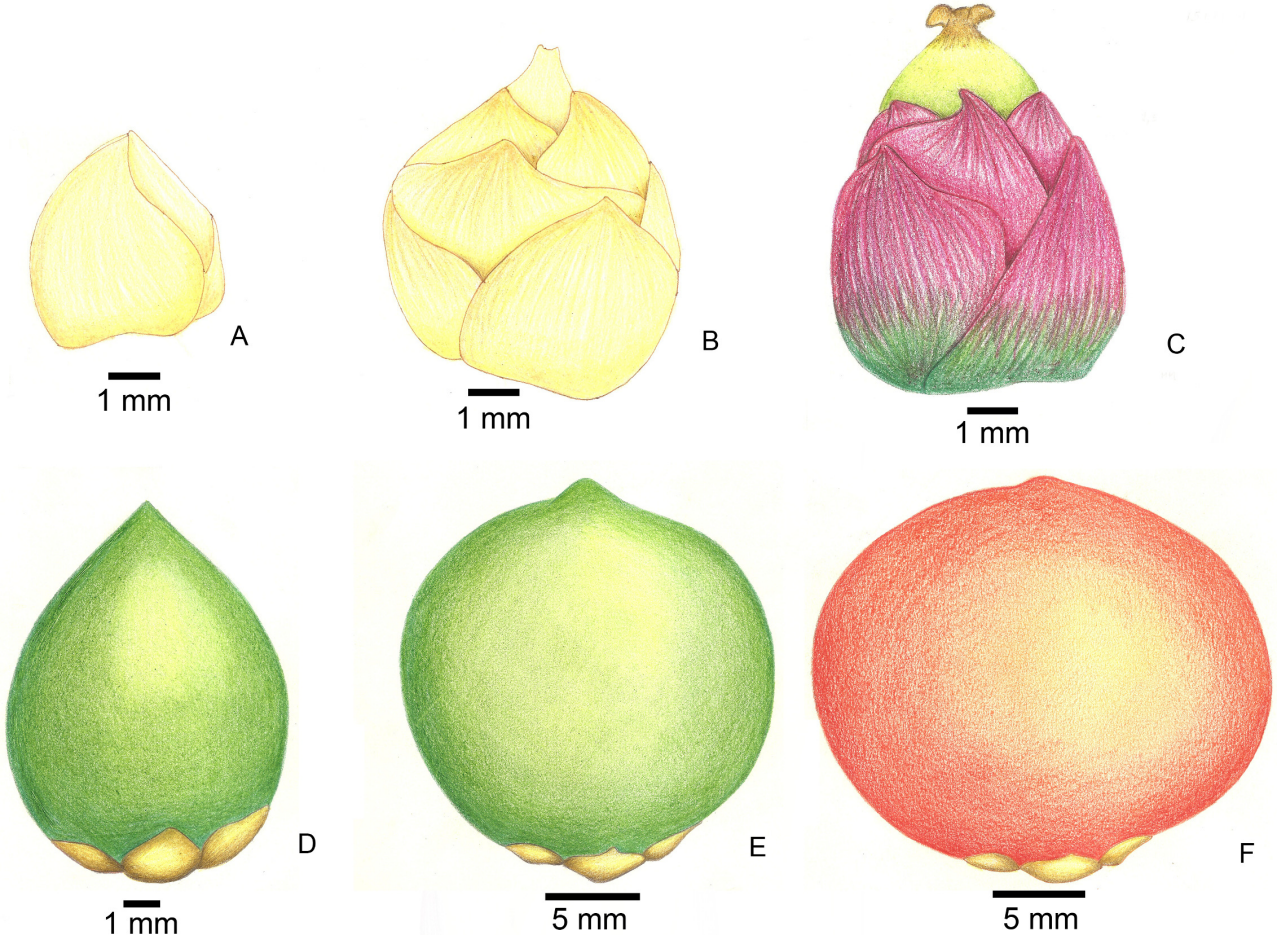
Stages of fruit development in *Butia eriospatha*. (A) Closed female flower, right after the spathe opens and flowers are exposed. (B) Opening female flower following male anthesis. Oviposition starts at this phase. (C) Young fruit a few days after pollination. (D) Older fruit before hardening of the endocarp. Some eggs are still laid during this phase. (E) Maturing fruit with hard endocarp. Larvae of *A. eriospathae* cannot penetrate fruits that reach this phase. (F) Ripe fruit.

Between October 2011 and January 2012, we collected samples from inflorescences in different stages (from open female flowers to young fruits, Fig. 2). We marked those inflorescences and repeatedly visited them 2–3 times per week to collect new larvae by cutting a few rachillae in each visit. Larvae and pupae were also searched in the soil surrounding trees and in the decaying material that accumulates on the persistent bases of old leaves. In the laboratory, rachillae were cut into several pieces and placed over moist filter paper in closed petri dishes. Larvae were reared in the lab and observed daily mostly to obtain specimens for studying morphology and behavior, and we recorded instar durations for a subset of the individuals. Since temperature and humidity were not controlled or recorded, developmental times reported here should be seen as guidelines and probably vary with weather conditions.

### Morphology

Live and fixed larvae were observed under a stereomicroscope and illustrated with the help of a *camera lucida* attached to a Leitz microscope Zeiss SM-Lux or a Wild stereomicroscope M5A. Larvae were fixed by boiling in water for a few seconds followed by preservation in ethanol 70%. To illustrate mouthparts, we made temporary preparations with glycerin in excavated slides. Black-and-white illustrations were inked in tracing paper and colored illustrations were prepared with color pencil. Illustrations were then scanned and processed in Adobe Photoshop CS3. Measurements were taken with an eyepiece micrometric scale attached to a stereomicroscope. Photos were taken using a camera attached to a stereomicroscope or an Automontage system (Leica M125 stereomicroscope coupled to digital camera Leica DFC). Terminology and abbreviations in larval and pupal descriptions follow Marvaldi (1999) and Oberprieler, Anderson & Marvaldi (2014).

After preliminary trials transplanting larvae to larger fruit resulted in larger adults, we decided to test the relationship between host plant fruit size and adult body size. We measured the length and width of the adult pronotum and the width of the ovaries from which they emerged for the first 50 adults to emerge starting on 13/VII/2012. Those individuals were collected as eggs between November and December 2011, from fruit still attached to their host plants. We used the geometric mean of pronotum length and width as a proxy for body size and correlated that with ovary size. Additionally, we fitted a linear regression using fruit size as an independent variable.

### Behavior

We observed the egg-laying behavior in the field, and most larval behaviors in the lab. We opened infested fruits or flowers and placed another larva to observe their interaction. This was done for each combination of different instars, repeated five times for each combination. To test the specificity of the response of *A. eriospathae*, we also tested interacting first-instar larvae with larvae of the pineapple beetle *Urophorus humeralis* (J. C. Fabricius, 1798) (Coleoptera, Nitidulidae), a pest on ripe fruits. Larvae of *U. humeralis* never meet larvae of *A. eriospathae* in nature, but they quickly bite each other and other larvae upon contact without causing any obvious damage (DC Bená, pers. obs., 2012).

### Taxonomy

Several adults of *A. eriospathae* and *A. hatschbachi* were observed to establish the synonymy between the two species. Abbreviations for the collections visited follow Evenhuis (2014):

• AMNH—American Museum of Natural History, New York, USA.
• DZUP—Museu de Entomologia Pe. Jesus Santiago Moure, Universidade Federal do Paraná, Curitiba, Brazil.
• CEAH—Coleção Entomológica Adolph Hempel, Instituto Biológico, São Paulo, Brazil.
• MLPA—Museo de La Plata, Universidad Nacional de La Plata, La Plata, Argentina.
• MNRJ—Museu Nacional, Universidade Federal do Rio de Janeiro, Rio de Janeiro, Brazil.
• MZSP—Museu de Zoologia, Universidade de São Paulo, São Paulo, Brazil.

The electronic version of this article in Portable Document Format (PDF) will represent a published work according to the International Commission on Zoological Nomenclature (ICZN), and hence the new names contained in the electronic version are effectively published under that Code from the electronic edition alone. This published work and the nomenclatural acts it contains have been registered in ZooBank, the online registration system for the ICZN. As of the date of publication of this work, ZooBank has not implemented registration of new synonyms. The ZooBank LSIDs (Life Science Identifiers) can be resolved and the associated information viewed through any standard web browser by appending the LSID to the prefix “http://zoobank.org/”. The LSID for this publication is: urn:lsid:zoobank.org:pub:D9C753ED-4A04-48B8-AC16-43C63CD5E254. The online version of this work is archived and available from the following digital repositories: PeerJ, PubMed Central and CLOCKSS.

## Results

### Descriptions

#### Fourth (last) instar larva (Figs. 3–5 and 9E)

##### Diagnosis

Length: 6.6–9.5 mm; prothorax width: 1.6–2.1 mm. Subcylindrical , C-shaped, weakly curved dorso-ventrally. Head hypognathous, gula absent. Four pairs of frontal setae and two pairs of clypeal setae present. Mandibles cuneiform, with one large apical tooth and one small median tooth. Ventropedal lobe setae of thorax and abdomen short.

**Figure 3:**
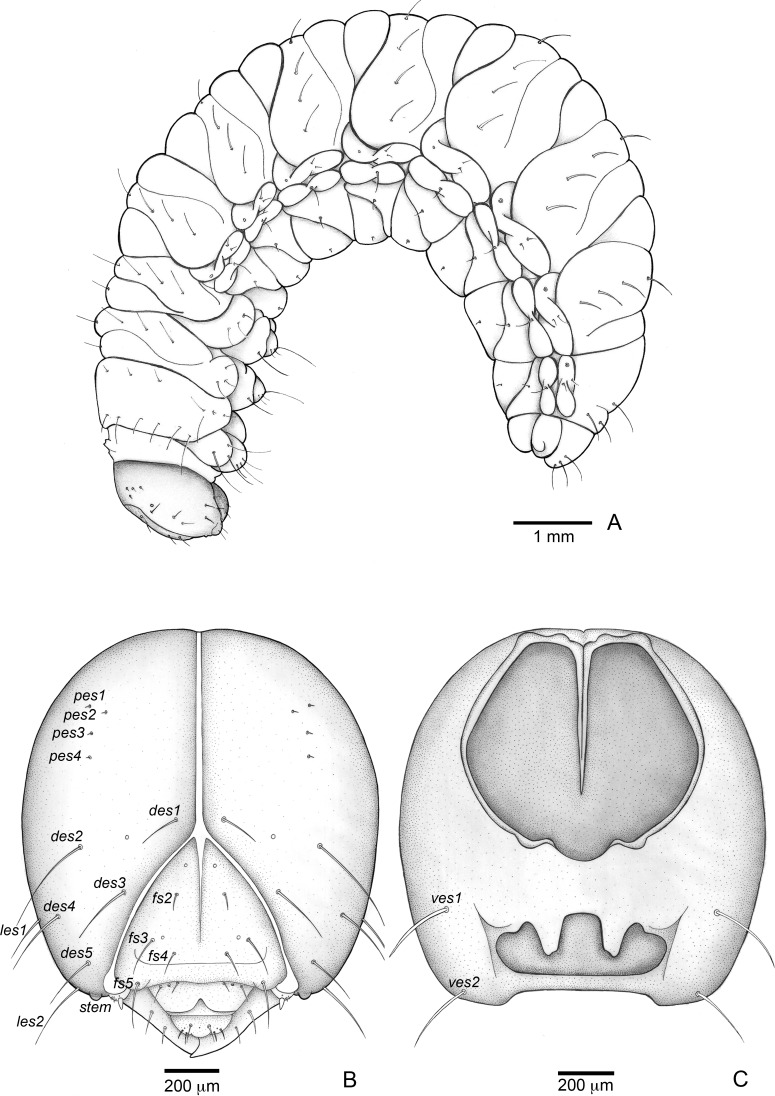
*Anchylorhynchus eriospathae*, fourth-instar larva. Habitus and head. (A) Habitus (lateral view). (B) Head capsule (frontal view). (C) Head capsule (posterior view). Abbreviations (s., seta or setae): *des*, dorsal epicranial s.; *fs*, frontal s.; *pes*, posterior epicarnial s.; *ves*, ventral epicranial s.; stem, stemma.

**Figure 4:**
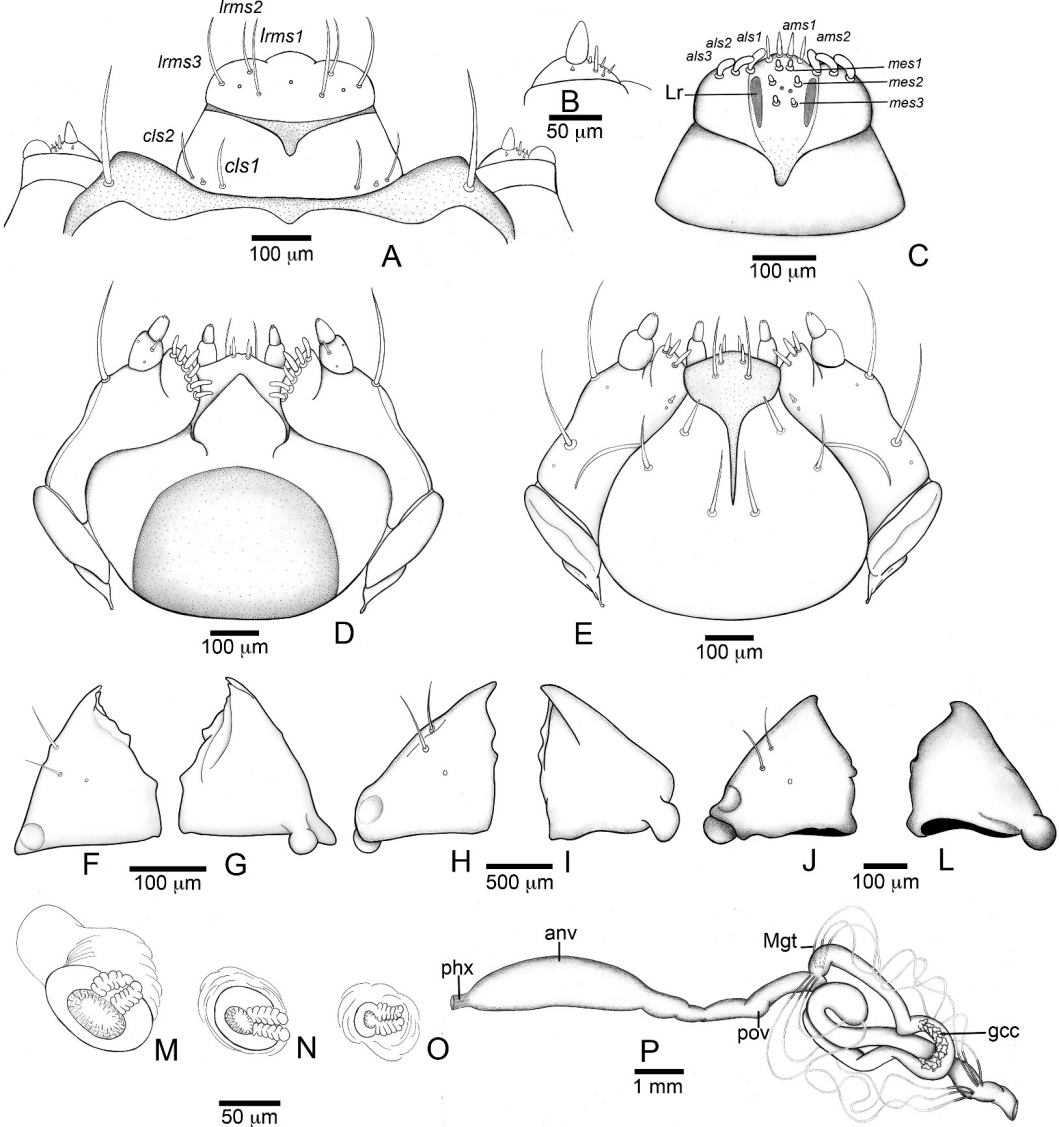
*Anchylorhynchus eriospathae* larvae. Mouthparts, spiracles and gut. (A–E), (J–P) Fourth instar, (F–G) second instar, (H–I) third instar. (A) Clypeus and labrum. (B) Antenna. (C) Epipharynx. (D) Maxillo-labial complex (dorsal view). (E) Maxillo-labial complex (ventral view). (F) Mandible (2th instar, dorsal view). (G) Mandible (2th instar, ventral view). (H) Mandible (3rd instar, dorsal view). (I) Mandible (3rd instar, ventral view). (J) Mandible (4th instar, dorsal view). (K) Mandible (4th instar, ventral view). (L) Prothoracic spiracle. (M) Abdominal spiracle I. (N) Abdominal spiracle VIII. (O) Alimentary canal. Abbreviations (s., seta or setae): *als*, anterolateral s.; *ams*, anteromedian s.; *anv*, anterior ventriculus; *cls*, clypeal s.; *gcc*, gastric caeca; *lrms*, labral s.; *mes*, median epipharyngeal s.; *Mgt*, Malpighian tubules; *phx*, pharynx; *pov*, posterior ventriculus.

**Figure 5:**
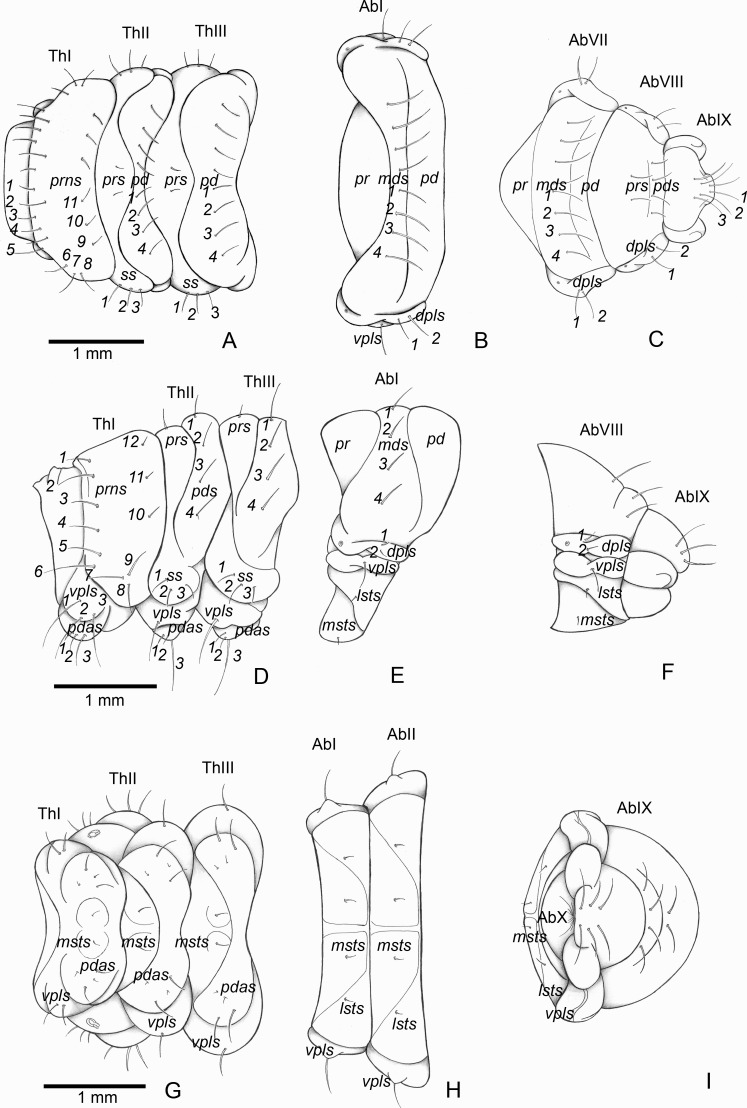
*Anchylorhynchus eriospathae*, fourth instar larva. Thorax and abdomen. Thoracic segments: (A) dorsal, (D) lateral, (G) ventral. Abdominal segment I: (B) dorsal, (E) lateral, (H) ventral. Abdominal segments VIII–IX: (C) dorsal, (F) lateral, (I) ventral. Abbreviations (s., seta or setae): *dpls*, dorsopleural s.; *lsts*, laterosternal s.; *msts*, mesosternal s.; *pdas*, pedal s.; *pds*, postdorsal s.; *prns*, pronotal s.; *prs*, pre dorsal s., *ss*, spiracular s.; *vpls*, ventropleural s.; *pd*, postdorsum; *pr*, predorsum; *Ab*, abdominal segment; *Th*, thoracic segment.

##### Description

Head (Figs. 3B and 3C). Hypognathous. Epicranium slightly longer than wide; coronal suture about 1/3 of epicranial length; frontal suture well developed, with 2/3 of frons length; endocarina present. One pair of stemmata, located laterally to antennal base. Fronto-clypeal suture simply curved not sinuous. Cephalic capsule with four pairs of minute epicranial posterior setae (*pes*), *pes2* not aligned with other three; five pairs of dorsal epicranial setae (*des*), *des1* and *des3* and *des5* located alongside frontal suture, *des2* and *des4* located more laterally, *des2* slightly longer than other four; four pairs of frontal setae (*fs*), *fs2* and *fs4* shorter than *fs3 and fs5, fs3 and fs4* do not surpass anterior margin of frons; two pairs of lateral epicranial seatae (*les1–2*); two pairs of ventral epicranial setae (*ves*), subequal. Clypeus (Fig. 4A) transverse, trapezoidal, posterior margin with two pairs of clypeal setae (*cls1–2*); one sensillum between *cls1* and *cls2*. Labrum (Fig. 4A) semicircular, with three pairs of labral setae (*lrms1–3*). Epipharynx (Fig. 4C) trapezoidal, anterior margin trilobate, median lobe with two pairs of spatulate anteromedial setae (*ams1–2*) and each lateral lobe with three pairs of larger spatulate anterolateral setae (*als1–3* curved inwards; labral rods weakly convergent backwards, stem absent; with three pairs of spatulate median epipharyngeal setae (*mes1–3*) and one pair of sensilla. Antennae (Fig. 4B) 1-segmented, with one elongate sensorial cone, bearing six minute sensilla, sensillum II longer and wider than others, III and IV similar sized, I and VI much smaller. Mandibles (Figs. 4J and 4K) cuneiform, symmetrical, with one large apical tooth and one small median tooth; with two dorsal setae. Maxillae (Figs. 4D and 4E): cardo elongate-oval, glabrous; stipes widened distally, ventrally with four sensilla: two in the outer margin, one located anteriorly and other posteriorly, and two very approximate in the inner margin; mala with a dorsal row of three spatulate setae and a ventral row of six aligned spatulate setae; palpi 2-segmented, palpomere I with three sensilla, one setiform. Hypopharynx (Fig. 4D). Ligula with triangular elevation. Labium (Fig. 4E): prementum sclerotized, with two pairs of setae, posterior pair about four times longer than anterior pair; mentum with three pair of setae, two lateral and one median; labial palpi 2-segmented, palpomere I as long as wide, palpomere II elongate, shorter and more slender than I. Gula absent.

Thorax (Figs. 5A, 5D and 5G). Pro-, meso- and metathorax transverse. Prothorax with 11 pairs of pronotal setae (*prns1–11*); ventropleural lobe with three pairs of setae (*vpls1–3*); pedal area with four pedal setae, 2 larger and two smaller (*pdas1–4*); mediosternal lobe unisetose (*msts1*). Meso- and metathorax: prodorsum with two setae (*prs*); postdorsum of meso- and metathorax with four pairs of postdorsal setae (*pds1–4*); dorsopleural lobe of meso- and metathorax with three pairs of setae (*dpls1–3*); ventropleural lobe of meso- and metathorax unisetose (*vpls*); pedal area with four pair of setae (*pdas1–4*), two large and two small; mediosternal lobe unisetose (*msts1*). Prothoracic spiracle (Fig. 4L) annular, biporous, with six oblique airtubes directed backwards.

Abdomen (Figs. 5B, 5C, 5E, 5F, 5H and 5I) 9-segmented; segments I–VII similar, with three dorsal, transverse plicae; segment VIII with two dorsal, transverse plicae; segment IX not plicate. Segments I–VII: postdorsal area with a transverse row of two pairs of postdorsal setae (*mds1–2*); mesodorsum with four pairs of mesodorsal setae *(mds1–4*); mediosternal area unisetose (*msts*); dorsopleural lobe with one pair of dorsopleural setae (*dpls1–2*); ventropleural lobe unisetose (*dpls1*); laterosternal lobe unisetose (*lsts*); mediosternal area unisetose (*msts*). Segment VIII: predorsum with one pair of setae (*prs*) and postdorsum with two pairs of setae (*pds1–2*). Segment IX reduced, trapezoidal, with three pairs of setae located posteriorly; laterosternal and ventropleural lobes very reduced. Segment X very reduced, elliptical, ventral. Anal slit terminal, surrounded by four fleshy lobes. Abdominal spiracles I–VIII (Figs. 4M and 4N) annular; biporous; airtubes with six annuli, spiracles I–VIII turned backwards.

Alimentary canal (Fig. 4O) lacking mycetomes; posterior ventriculus (*pov*) two coiled; with 20 short, papilliform gastric caeca (gcc), axially aligned forming two compact lines on lower ventricular coil; Malpighian tubules (*Mgt*) arranged 3 + 3.

#### Third instar larva (Figs. 4H and 4I)

Length: 6.2–7.0 mm; prothorax width: 1.5–1.6 mm.

#### Second instar larva (Figs. 4F, 4G and 9D)

Length: 3.0–5.1 mm; prothorax width: 0.9–1.1 mm.

##### Remarks

Second and third instar closely resemble fourth instar larvae, and share very similar chaetotaxy. Body milky white, with a few short and fine setae.

#### First instar larva (Figs. 6, 7, 9B and 9C)

##### Diagnosis

Length: 1.5 mm; largest prothorax width: 0.7 mm. Body strongly flattened. Head prognathous, about one fourth of body length, gula present. Three pairs of frontal setae present. Clypeal setae absent. Mandibles falciform with serrate retinaculum. Ventropedal lobe setae of thorax and abdomen very elongate, about as wide as prothorax width.

**Figure 6:**
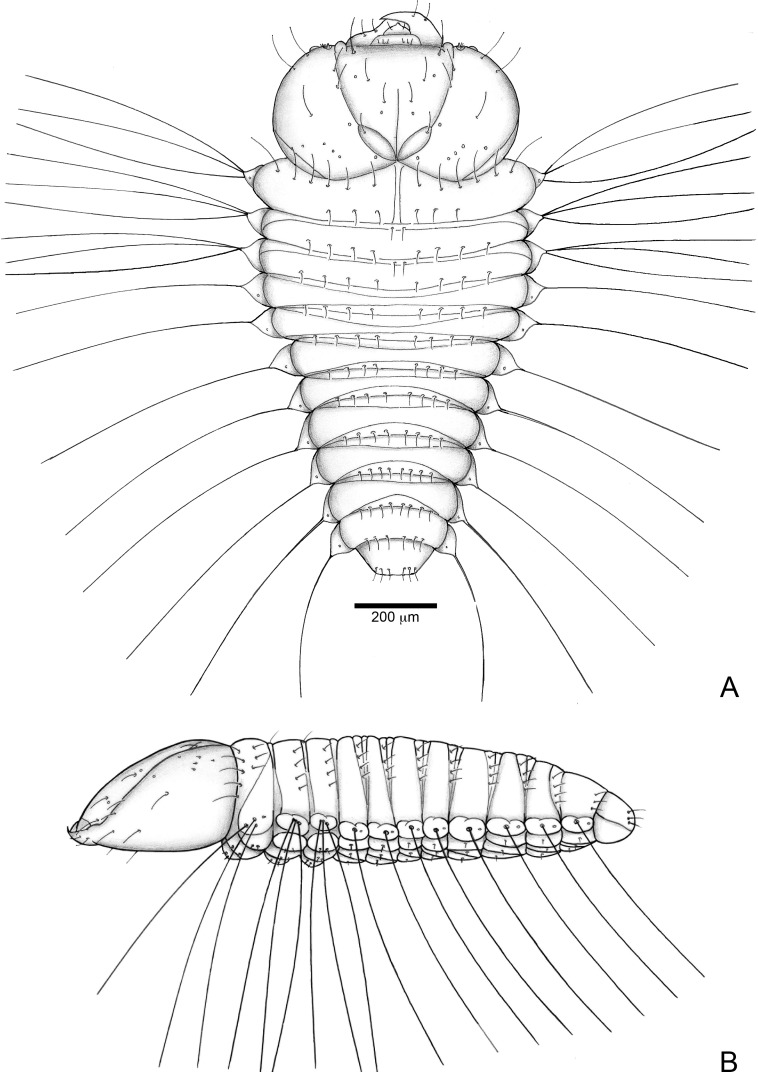
*Anchylorhynchus eriospathae*, first-instar larva. Habitus. (A) Dorsal view. (B) Lateral view.

**Figure 7:**
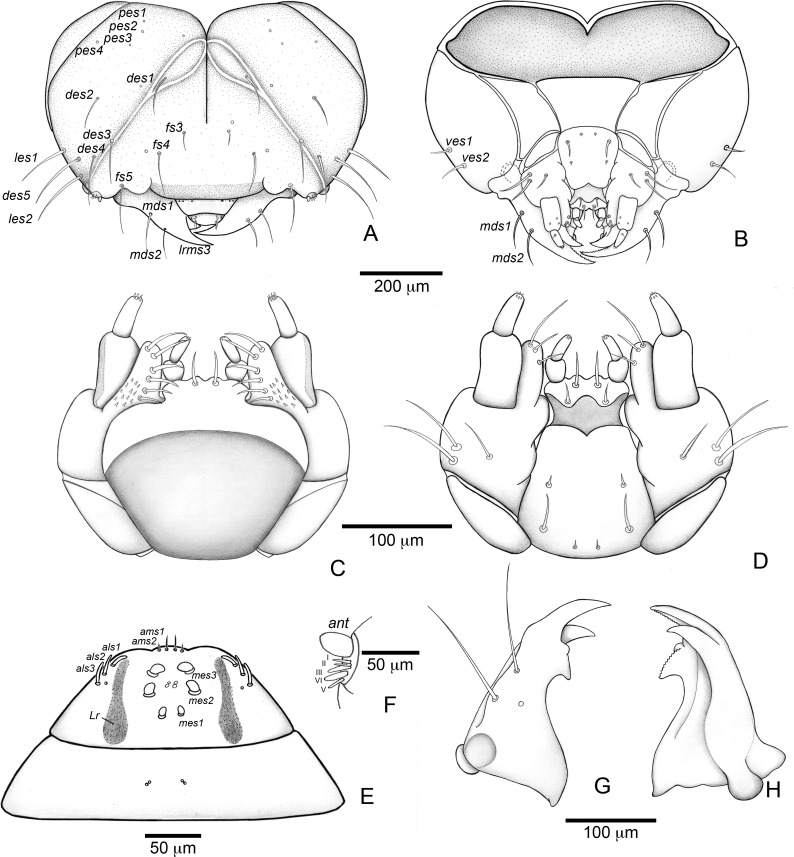
*Anchylorhynchus eriospathae*, first-instar larva. Head and mouthparts. (A) Head capsule (frontal view). (B) Head capsule (posterior view). (C) Maxillo-labial complex (dorsal view). (D) Maxillo-labial complex (ventral view). (E) Antenna. (F) Epipharynx. (G) Mandible (dorsal view). (H) Mandible (ventral view). Abbreviations (s., seta or setae): *als*, anterolateral s.; *ams*, anteromedian s.; *ant*, antenna; *des*, dorsal epicranial s.; *fs*, frontal s.; *les*, lateral epicranial s.; *Lr*, labral rods; *lrms*, labral s.; *mes*, median epipharyngeal s.; *mds*, mandibular s.; *pes*, posterior epicarnial s.; *ves*, ventral epicranial s.

**Figure 8:**
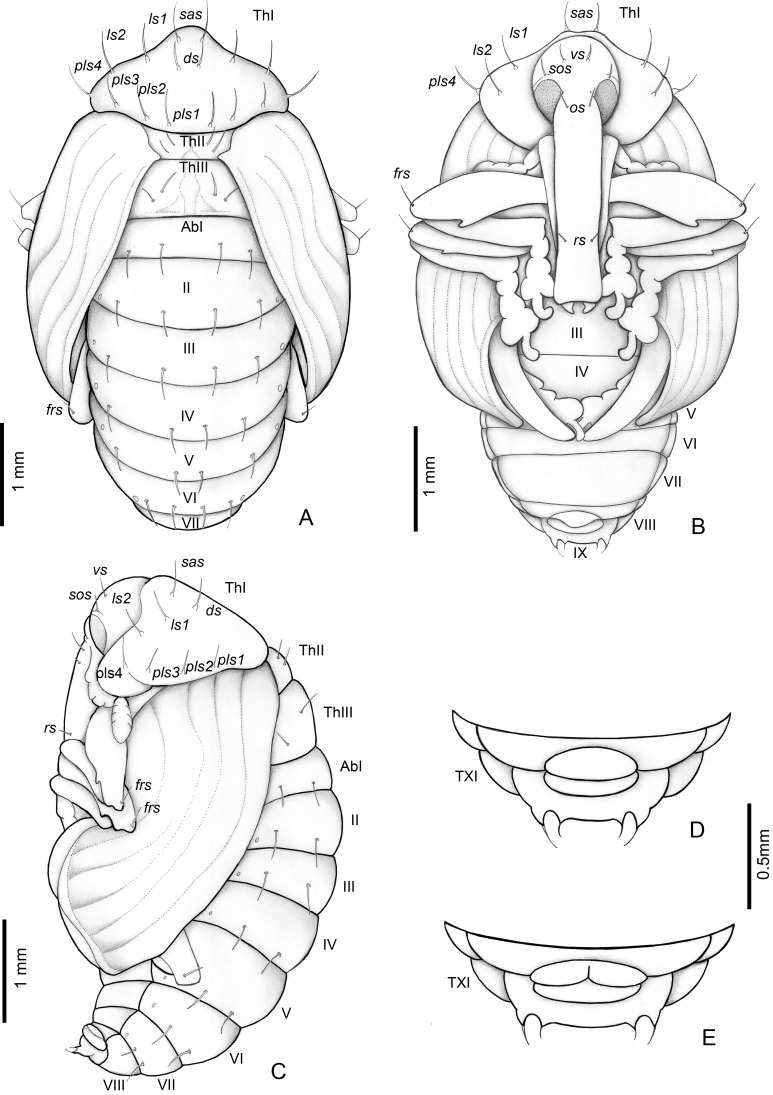
*Anchylorhynchus eriospathae*, pupa. Habitus: (A) dorsal view, (B) ventral view, (C) lateral view. Abdominal extremity, ventral view: (D) male, (E) female. Abbreviations (s., seta or setae): *ds*, discal s.; *frs*, femoral s.; *ls*, lateral s.; *os*, orbital s.; *pls*, posterolateral s.: *rs*, rostral s.; *sas*, super apical s.; *sos*, superorbital s.; *vs*, vertical setae; *Th*, thoracic tergite; *Ab*, abdominal tergite.

**Figure 9:**
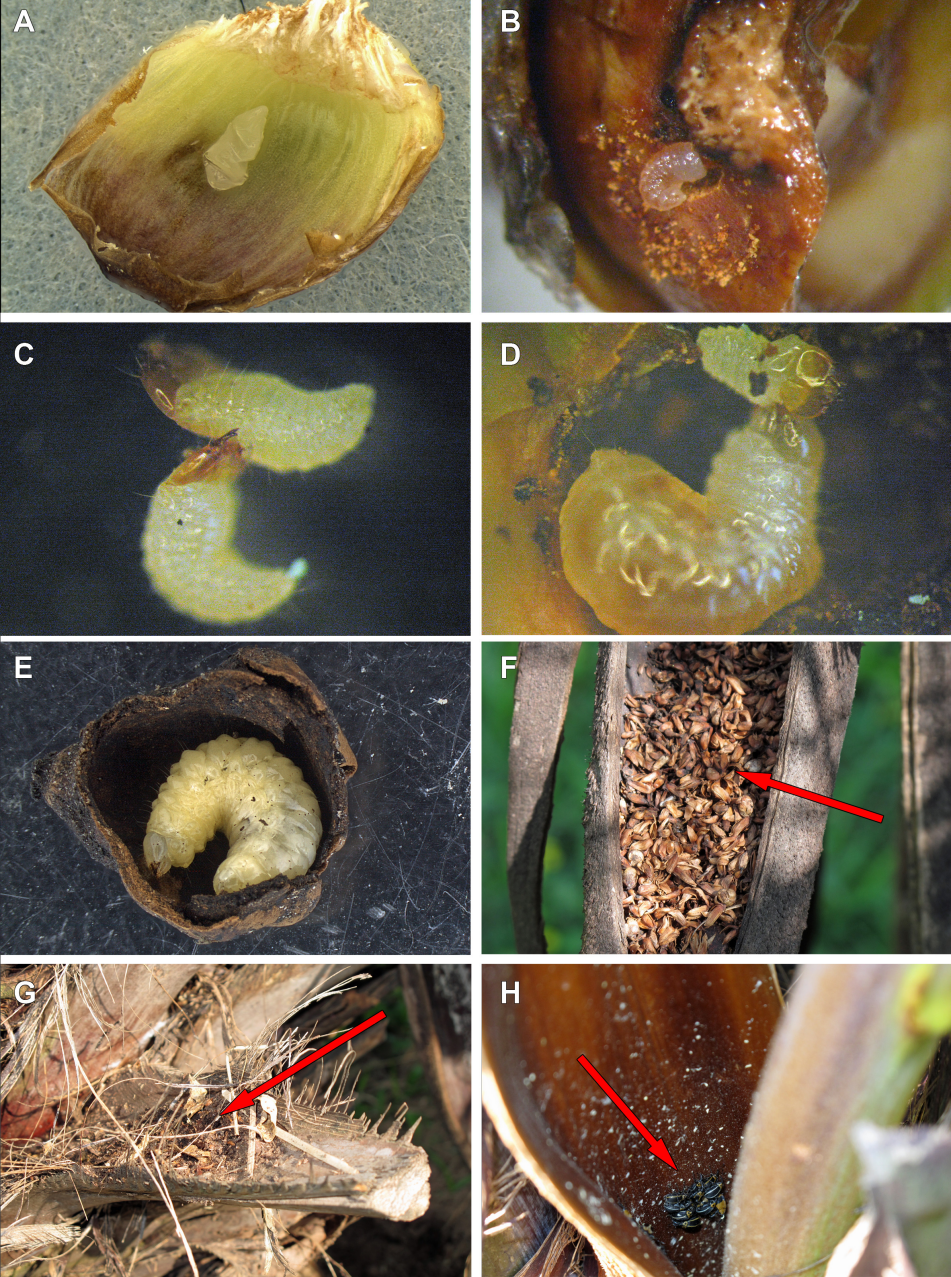
Life history of *Anchylorhynchus eriospathae.* (A) Recently laid egg over a bract (transversal folds are an artifact of manipulation). (B) First-instar larva drilling the base of a fruit. (C) First-instar larva attacking another first-instar larva. (D) Second-instar larva attacking a first-instar larva. (E) Fourth-instar larva after having consumed almost all the internal part of a fruit. (F) Aborted fruits and flowers (arrow) trapped by old peduncular bract. (G) Persistent leaf bases trapping aborted flowers and fruits (arrow). (H) Adults of *A. eriospathae* (arrow) hiding at the base of an inflorescence.

##### Description

Head (Figs. 7A and 7B) Prognathous, about 1.6 times as wide as long. Cephalic capsule deeply emarginate behind. Coronal suture absent; frontal suture about 4/5 as long as epicranial length, subdivided in basal third forming two elliptical areas; endocarina present, with 1/2 of frons length; fronto-clypeal suture present; *pes4* inserted laterally, not aligned with other *pes* (*pes3–4*); five pairs of dorsal epicranial setae (*des1–5*), *des1* located in elliptical frontal area, *des3* and *des4* located alongside frontal suture, *des2* and *des5* located more laterally, *des5* slightly longer than other four; with three pairs of frontal setae (*fs3–5*), *fs3* smaller, fs4 and fs5 subequal, fs5 surpass anterior margin of labrum; two pairs of lateral epicranial setae (*les1–2*); with two pairs of ventral setae (*ves1–2*), *ves2* slightly longer than *vs1.* Clypeus without clypeal setae. Labrum (Fig. 7A) trapezoidal, with one three pairs labral of setae (*lrms1–3*), *lrms1* very short, *lrms1* and *lrms2* longer and subequal. Epipharynx (Fig. 7E) trapezoidal, anterior margin trilobate, median lobe with two pairs of anteromedial setae (*ams1–2*), *ams1* longer, and each lateral lobe with three pairs of slender spatulate anterolateral setae (*als1–3*) curved inwards; labral rods subparallel, stem absent; with three pairs of spatulate median epipharyngeal setae (*mes1–3*) and one pair of sensilla. Antenna (Fig. 7F) 1-segmented, with elongate sensorial cone and five spatulate elongate sensilla: I, II and V thinner, III and IV larger and broader. Mandibles (Figs. 7G and 7H) falciform, elongate, symmetrical, with one apical (dorsal) and one subapical (ventral) teeth, and one posterior, triangular-shaped retinaculum (dorsal); inner ventral margins of dorsal tooth and retinaculum serrate. Maxillae (Figs. 7C and 7D) stipes with three long setae in ventral view (Fig. 7D); mala, in dorsal view (Fig. 7C), with spiniform projections located in basal half. Hypopharynx (Fig. 7C) with sinuous anterior margin. Labium (Fig. 7D): prementum with two pairs of setae, posterior pair slightly longer than anterior pair. Gula present (Fig. 7B), trapezoidal, transverse.

Thorax (Fig. 6). Pro-, meso- and metathorax transverse, flattened.

Abdomen (Fig. 6). Segments I–IX flattened. Segment VIII with one transverse row of three setae. Segment IX reduced, trapezoidal, with one transverse row of six setae; laterosternal and ventropleural lobes ventralized and reduced. Segment X very reduced, circular, terminal.

##### Remarks

The first instar larva is very different from the fourth instar, and is well characterized by the diagnosis and description presented above. The chaetotaxy is also very similar to the fourth instar, other than in the setae arrangement detailed in the description. It is noteworthy that all clypeal setae are absent–an unusual arrangement within Curculionidae. Three rounded structures are visible, but it is unclear whether they represent sensilla or sockets.

#### Pupa

(Fig. 8).

##### Description

Length: 4.5–3 mm; largest 3–5.1 mm. Adecticous and exarate. Body, including setae and spines, light yellow. Head covered by pronotum in dorsal view; each side with one vertical setae (*vs*), one supraorbital setae (*sos*), one orbital setae (*os*) and one rostral setae (*rs*). Rostrum surpassing anterior margin of abdominal segment IV. Rostrum of males and females of similar length.

Pronotum tranverse, triangular-shaped, anterolateral margins strongly sinuous, posterior margin strongly curved; each side of pronotum with one discal seta (*ds*), one suprapical seta (*sas*), two pairs of lateral setae (*ls1–2*), and four posterolateral setae (*pls1–4*). Mesothorax with two fine and erect setae on each side, located on disc. Scutellum semicircular. Metathorax with a dorsal median longitudinal sulcus, which divides the discal area in two halves, each with two tergal setae. Abdomen: abdominal segments I–VII with four tergal setae forming a transverse row near posterior margin; segments VIII and IX reduced, segment IX only visible in ventral view, with pseudocerci (*pc*), each one with one pair of micro-setae inserted on small, acute processes. Gonotheca divided in females (Fig. 8E), not divided in males (Fig. 8D). Pterotheca extending up to apex of fifth abdominal ventrite (Fig. 8B). Abdomen with seven pairs of annular spiracles.

### Life history and behavior

#### Oviposition

Females lay eggs in female flowers. Oviposition starts as soon as female flowers begin to open during male anthesis and continues until the endocarp becomes hard. For that reason, we will use the term “ovary” here to refer interchangeably to flowers or fruits consumed by larvae along this continuum. Instead of drilling a hole into the ovary with the rostrum as many species of Curculionidae do (Oberprieler, Marvaldi & Anderson, 2007), females of *A. eriospathae* place the eggs externally between the bracts that surround the gynoecium (Video S1). Most flowers have approximately three eggs, usually in different stages of development. Presumably, they were laid on different days, but we do not know if by the same female. While most eggs are deposited between bracts some were found underneath the bract cuticle.

#### Egg

Eggs are flat, elliptic, slightly curved following the bract curvature (Fig. 9A). This stage lasts approximately 11 days at room temperature (10 eggs observed) , but we recorded up to 16 days in the field. Newly laid eggs are transparent, gradually becoming white after four days. By the fifth day, sclerotized mandibles are observable and by the ninth day the head is distinct. The larva seems to be fully formed by the tenth day. Approximately four hours prior to hatching, larvae begin to undulate, moving forward inside the egg. When mandibles touch the eggshell, the body movement stops and they start to move the mandibles, rupturing the egg. Once the eggshell is ruptured, the larva slowly moves forward through the hole.

#### First instar

This stage lasts approximately 3–3.5 days (10 larvae observed). After leaving the egg, the larva remains immobile for approximately three hours. After that, they do not consume the eggshell and instead rapidly move towards the base of the ovary. They slide between bracts and do not damage them. Most encounters between first-instar larvae take place while they move towards the base. Once a larva reaches the base of the flower or fruit, it drills a hole towards the seed (Fig. 9B). The larvae molt as soon as the gallery is large enough to hold them, and exuviae can later be found in those galleries.

We offered eggs, first-instar larvae, second-instar larvae and larvae of pineapple beetles to first-instar weevil larvae. The weevils ignored eggs and pineapple beetles, even when bitten by the latter. They usually avoided second-instars, moving away from them and never attacking. Finally, first-instar larvae immediately attack conspecifics of the same instar (Fig. 9C). Following a successful attack (Video S2), they consume the killed larvae in approximately 20 min. We did not observe any obvious relationship between larval size or age and attacking success.

#### Second instar

**Figure 10:**
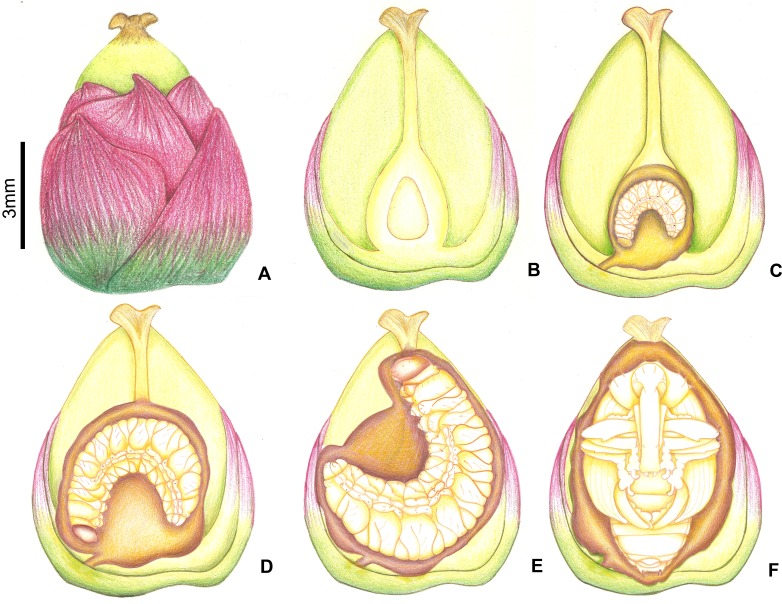
Growth of immatures of *A. eriospathae* inside fruits of *B. eriospatha*. (A) External view of a recently fertilized fruit. (B) Internal view of a non-infested fruit. (C) Second-instar larva. (D) Third-instar larva. (E) Fourth-instar larva. (F) Pupa.

The second instar lasts approximately 3.5–4 days (10 larvae observed), and the larvae consume up to a third of the volume of the ovary (Fig. 10). Most of the larvae were found inside ovaries still attached to the inflorescence, although some second-instar larvae were found in aborted ovaries. We found only one second-instar larva per flower, but larger fruits sometimes were shared with other larvae. However, we sometimes did find a second-instar larva sharing a large non-aborted fruit with another second-instar or a third-instar larva. They would occupy opposite ends inside the fruit, seemingly to avoid contact with each other. Second-instar larvae are also able to colonize new fruits following abortion by making galleries between aborted fruits directly in contact with each other.

We offered larvae from first to fourth instar and larvae of pineapple beetles to second instar larvae, and they only attacked and cannibalized first-instar larvae (Fig. 9D, Video S3). As soon as first-instar larvae approached their galleries, second-instar larvae attacked and consumed them.

#### Third instar

The third instar lasts approximately five days (2 larvae observed). Third-instar larvae continue feeding on the ovary, consuming approximately two thirds of its volume (Fig. 10). We found a single larva in most fruits, although sometimes large non-aborted fruits were shared with second-instar larvae. Third-instar larvae were found both in aborted and non-aborted fruits. They are not aggressive and seem to avoid contact with other larvae.

#### Fourth instar

Fourth-instar larvae continue feeding on the internal ovary tissues, leaving only a thin layer when finished (Figs. 9E and 10). Most of them are found in the aborted ovaries. Unlike the other instars, the fourth instar duration is highly variable. Once larvae finish feeding, they remain immobile for at least 30 days (80 larvae observed). Some larvae remained in this stage for more than 120 days. They do not enter diapause, as they immediately respond when disturbed. Prior to pupation, larvae empty their guts and change color from translucent creamy-yellow to opaque yellow. Fourth-instar larvae are not aggressive and avoid contact with other larvae.

#### Pupa

Pupation takes place inside the consumed flower/fruits (Fig. 10), many of them found in the persistent leaf bases, the base of the inflorescence or in old peduncular bracts (Figs. 9F and 9G). We have not found pupae in the soil surrounding trees. This stage lasts 5–8 days.

#### Adult

Adults are diurnal and spend most of the time hidden at the base of inflorescences, resting and mating (Fig. 9H). They visit male flowers and feed on pollen from open and closed male flowers (Video S4). In the laboratory, we could keep them alive for up to 16 days, if reared among freshly cut flowers that they used for feeding.

### Body size

**Figure 11:**
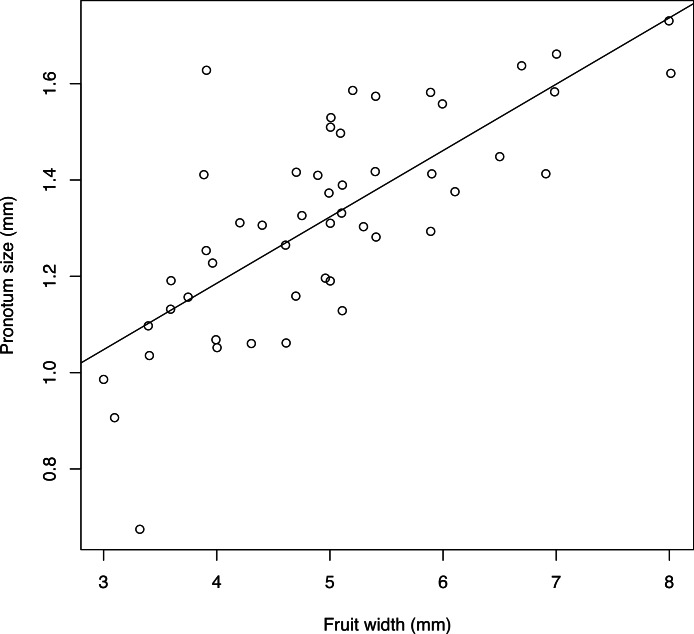
Relationship between fruit width and size of emerged adult. Size of adults is the geometric mean between pronotum length and pronotum width. Line indicates the linear regression. A small random jitter (normal with mean 0 and standard deviation 0.007) was added to each point to enable visualization of overlapping records.

Body size and fruit width are strongly correlated (Pearson correlation 0.746, *p* < 0.001). The regression coefficient was highly significant (value = 0.1378, *p* < 0.001), showing a clear linear relationship between fruit size and body size (Fig. 11).

### Taxonomy

**Figure 12:**
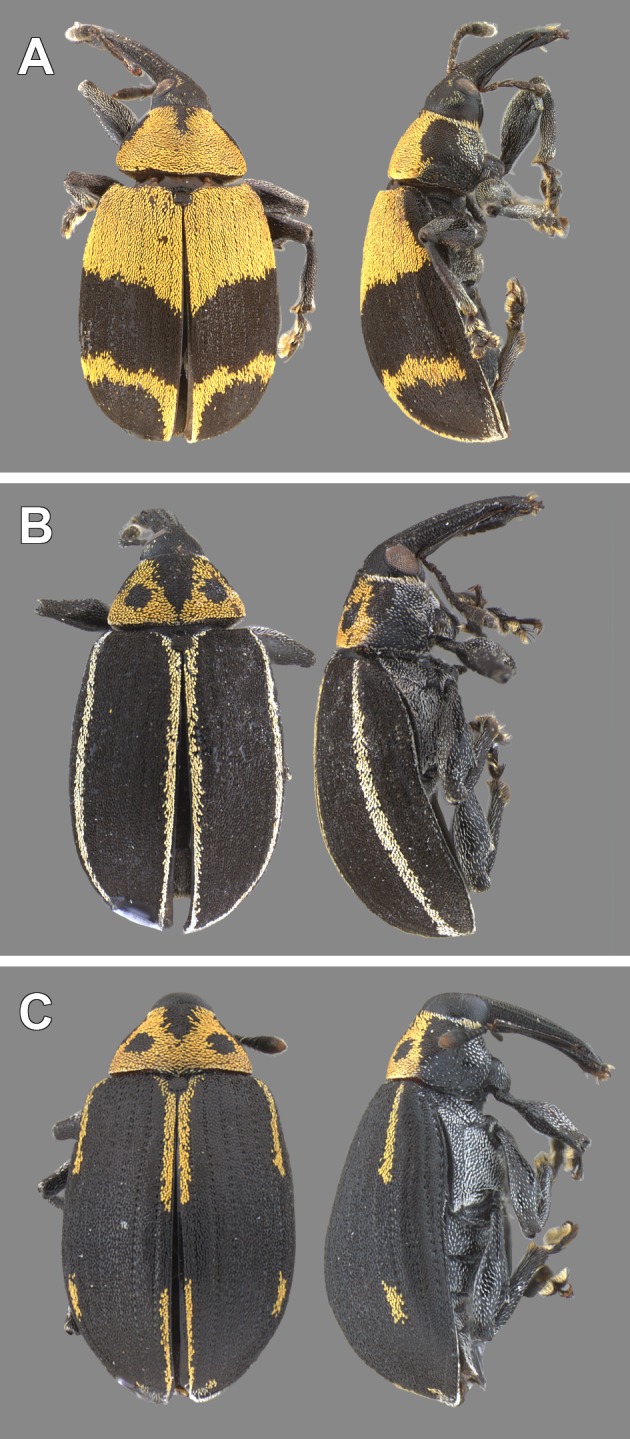
Color morphs of adults of *Anchylorhynchus eriospathae* found in Cidade Universitária. As defined by Bondar (1943), *Anchylorhynchus hatschbachi* (junior synonym) has either the pattern shown in (A) or uniformly yellow dorsal surface of the elytra, and *Anchylorhynchus eriospathae* has the pattern shown in (B) or additional yellow stripes on intervals 3 and 5. (C) color pattern not described in the literature, intermediate between *A. hatschbachi* and *A. eriospathae*.

Bondar (1943) described *A. eriospathae* and *A. hatschbachi* from specimens collected from a single inflorescence of *Butia eriospatha*. The only difference between adults of the two species is the color pattern of the pronotum and the elytra (Figs. 12A and 12B). Even though he acknowledged they could represent color morphs of the same species and both color morphs are often found together (see examined material below), a more recent taxonomic revision maintained the species status (Vaurie, 1954). Here we found the two color morphs together in the same individual trees, including undescribed intermediate morphs (Fig. 12C). Additionally, we could not detect any difference in the larval morphology and behavior, or in the adult genitalia. We conclude therefore, that *A. eriospathae* and *A. hatschbachi* are the same species and should be synonymized. Since both were described in the same article, there is no priority, and we choose *Anchylorhynchus eriospathae* as the senior synonym because the name is biologically more informative.

#### Material examined

##### Specimens formerly identified as *A. eriospathae*

Lectotype: Male. Brazil, Curitiba, Paraná, deposited in the AMNH (examined).

BRAZIL. **Paraná**: Curitiba, B Pohl, 1/I/1943 (1 female (MZSP)); same but Casagrande, 17/XII/1980 (1 male (DZUP)); same but 27/XII/1980 (4 female (DZUP)); same but illegible coll., I/1944 (1 sex unobserved paralectotype (MLPA)); same but no collector, I/1946 (1 male, 1 female (AMNH)); same but no date (2 male, 3 female (AMNH); 1 female paralectotype (IB); 2 female paralectotype (MNRJ)); **Rio Grande do Sul**: Canela, M Hoffman, 22/XI/1990 (1 male (DZUP)); Xanxerê, no collector, XI/1977 (1 male (DZUP)); **Santa Catarina**: Nova Teutônia, F Plaumann, XI/1977 (1 male, 2 female (DZUP)); **São Paulo**: Cidade Universitária, São Paulo, BAS Medeiros, 6/X/2009 (2 female (MZSP)); same but 7/X/2009 (1 female (MZSP)); São Paulo, Fabiana D’Agostino, XI/2014 (3 male, 5 female (MZSP)); no label (1 female (CEAH)).

##### Specimens formerly identified as *A. hatschbachi*

Lectotype: Male. Brazil, Curitiba, Paraná, deposited in the AMNH (examined).

BRAZIL. **Paraná**: Curitiba, A Maller, no date (2 male paralectotype, 3 female paralectotype (MNRJ)); same but B Pohl, I/1943 (2 male paralectotype (MZSP)); same but Casagrande, 27/XII/1980 (7 female (DZUP)); same but illegible coll., I/1944 (3 female paralectotype (MLPA)); same but no collector, 1943 (1 male, 1 sex unobserved (CEAH)); same but no date (6 male paralectotype, 6 female paralectotype (AMNH); 2 female paralectotype (MLPA)); **Rio Grande do Sul**: Xanxerê, no collector, XI/1977 (11 sex unobserved (DZUP)); **Santa Catarina**: Cauna, A Maller, I/1946 (7 male, 4 female (AMNH)); Corupá, A Maller, XI/1945 (1 male, 3 female (AMNH)); same but XI/1946 (2 male, 3 female (AMNH)); Lages, SS Ortolan, XII/1981 (1 male, 2 female (DZUP)); Nova Teutônia , F Plaumann, XI/1977 (8 male, 5 female (DZUP)); Rio Vermelho, Dirings, VIII/1950 (37 male, 36 female (MZSP)); São Bento do Sul, Dirings, II/1951 (33 male, 43 female (MZSP)); **São Paulo:** Cidade Universitária, São Paulo, BAS Medeiros, 6/X/2009 (1 male (MZSP)); same but 7/X/2009 (1 female (MZSP)); same but 18/XI/2009 (1 female (MZSP)); São Paulo, Fabiana D’Agostino, XI/2014 (3 male, 3 female (MZSP)). No locality, C. O., 17/XII/1937 (1 female (MZSP)); no label (1 male, 1 female (CEAH)).

## Discussion

The peculiar morphology of the first instar larva of *A. eriospathae*, unique in the Curculionoidea, is an evident adaptation to a particular way of life. They slide between the sepals and petals of the female flower bud, mature flower, or developing fruit and only after the molt, the second subcylindrical instar digs deep inside the ovary tissue. The proportionately large and prognathous head, with a gula, has powerful muscles that move the falciform mandibles. These slender structures are adapted to predation, have a serrate retinaculum, and are used to fight with and eventually kill other first-instar larvae. Finally, the very long ventropedal lobe setae of thorax and abdomen are probably important structures able to detect the approximation of conspecific larvae.

First-instar larvae are even more distinct than those of *Revena rubiginosa*, in which the shape of the mandibles was the main difference between first- and late instars (Alves-Costa & Knogge, 2005). *Anchylorhynchus* (Curculioninae: Derelomini *sensu* Caldara, Franz & Oberprieler (2014)) and *Revena* (Conoderinae: Bariditae) belong to very distinct groups in Curculionidae, and it seems that falcate mandibles in larvae are not common in either taxon.

Very few larvae of Bariditae have been described so far, but Pakaluk (1993) listed 13 genera of Bariditae that had some larval stage described and we found descriptions of eigth additional genera since then (Nikulina, 2012; Epsky et al., 2008; Ulmer et al., 2007; Pakaluk, 1994). Falcate mandibles are not mentioned in any description. In most cases, it is not clear which larval stage was described, but in a few cases the authors claim to have followed the development from the egg stage (Epsky et al., 2008; Ulmer et al., 2007). In those cases, we can infer that first-instar larvae were unexceptional.

*Anchylorhynchus* is the first genus of Derelomini for which a detailed description of larvae is available. The fourth-instar larva of *A. eriospathae* agrees very well with the diagnosis of the Curculioninae larvae reported by May (1994) and Caldara, Franz & Oberprieler (2014). It is important to highlight that Curculioninae is likely a polyphyletic assemblage (McKenna et al., 2009), so more meaningful comparison should be done with Derelomini, which is probably monophyletic (Franz, 2006). In spite of the lack of proper descriptions, several authors claim to have observed larvae of other genera in the Derelomini (Tuo, Koua & Hala, 2011; Franz & Valente, 2005; Bondar, 1943), including studies that followed the development from the egg stage (Tuo, Koua & Hala, 2011; Franz & Valente, 2005). None of those studies mentions falcate mandibles or aggression between larvae. We have observed larvae of *Elaeidobius kamerunicus* (J. Faust, 1898) and *E. subvittatus* (J. Faust, 1898) (a genus closely related to *Anchylorhynchus* (Franz, 2006) of different sizes–presumably, different instars–and all of them seemed to have triangular mandibles. Even though more accurate studies are required, it is clear that the predatory morphology arose independently in *Anchylorhynchus* and *Revena*, and it seems that it is not a common feature among closely related genera of either group. The only other taxon with falcate mandibles in Curculionidae that we are aware of is *Ozopemon* M. Hagedorn, a genus in the subfamily Scolytinae. In this case, neotenic males have sclerotized heads with strong falcate mandibles used to kill other males (Jordal et al., 2002).

*Revena rubiginosa* lays its eggs at a later stage in fruit development if compared to *Anchylorhynchus*, right before the endocarp becomes hard. After that, adults cannot drill through the endocarp, so the surviving larva does not have any more competitors (Alves-Costa & Knogge, 2005). *Anchylorhynchus*, on the other hand, starts oviposition during flowering and adults do not drill flowers. There is intense competition between first-instar larvae for access to the ovary, but the flower/fruit continues to be accessible to new first-instar larvae after the first larva molts. When larvae of different ages share a large enough fruit, they seem to switch to a scramble mode of competition and avoid direct interference with each other. Contrary to what was suggested by the biology of *R. rubiginosa*, a specialized first-instar killer can evolve and/or be maintained even when later instars can experience competition with conspecifics.

The large time window of oviposition in *Anchylorhynchus eriospathae* also seems to be related to the great variation in body size in this species. Young and small flowers result in smaller individuals, suggesting that there are costs in being the first larva to occupy an ovary. On the other hand, the first occupants likely encounter and feed on more larvae, possibly complementing their diet. Further studies on this system could elucidate the trade-offs involved and the role of cannibalism in nutrition.

In spite of all particularities in the life histories of *Anchylorhynchus* and *Revena*, species in both genera and other closely related weevils co-inhabit the same plants. For example, three other species of *Anchylorhynchus* feed on female flowers of *Syagrus romanzoffiana* (Vaurie, 1954), the host of *Revena rubiginosa*, and first-instar larvae of *A. aegrotus*, *A. variabilis* and several other species of *Anchylorhynchus* superficially resemble those of *A. eriospathae* (B de Medeiros, pers. obs., 2011–2014). Adults of both *Anchylorhynchus* and *Revena* emerge from fruits of *S. romanzoffiana*, with *A. aegrotus*, *A. variabilis* and *R. rubiginosa* being found in the same individual plants (da Silva et al., 2012; B de Medeiros, pers. obs., 2014). Therefore, it is possible that larvae of all of the species encounter each other. In fact, seed beetles also develop inside fruits of *Syagrus* and other palms (Nilsson & Johnson, 1993), and we have observed larvae of bruchines attacking *B. eriospatha* as well. They are able to oviposit even later than *Revena*, and larvae enter the hard endocarp through the phloem (Bondar, 1937). The first-instar larvae of *Pachymerus cardo* (Fahraeus, 1839), a species that feeds on several palm species (including *S. rommanzofianna*), have mandibles that are more pointed than those of later instars (Prevett, 1968), but it is not known if they are used to kill other larvae. It is possible, therefore, that the morphologies of *Anchylorhynchus*, *Revena*, *Pachymerus* and other still unstudied beetles evolved in a complex scenario of intra- and inter-specific competition.

If cannibalism and contest competition is indeed common among concealed herbivores, it is possible that killer phenotypes at some larval stage are more common than currently acknowledged. In order to study that, we need to properly document the morphology and behavior of a greater diversity of larvae, including the often-overlooked first instars. With a greater knowledge of the frequency of killer morphologies among herbivores, we would be better able to understand the trade-offs involved and the ecological contexts in which they evolve.

## Supplemental Information

10.7717/peerj.502/supp-1 Video S1 Oviposition in *Anchylorhynchus eriospathae* A female of *Anchylorhynchus eriospathae* laying an egg on a female flower of *Butia eriospatha* during male anthesis (*B. eriospatha*) is protandrous. Notice that the female does not drill with the rostrum and instead lays the egg between the bracts that surround the flower.

10.7717/peerj.502/supp-2 Video S2 Cannibalism among first-instar larvae of *Anchylorhynchus eriospathae* We removed two first-instar larvae of *Anchylorhynchus eriospathae* and placed them in Petri dishes for better visualization. Here a larva consumes another that was recently attacked and still moves its mandibles.

10.7717/peerj.502/supp-3 Video S3 Second-instar larva of *Anchylorhynchus eriospathae* feeding on first-instar larva The first-instar larva was offered to a second-instar larva immediately after we opened the fruit. This video shows the bigger larva consuming the smaller one after killing it.

10.7717/peerj.502/supp-4 Video S4 Adult feeding in *Anchylorhynchus eriospathae* Adults visit inflorescences to feed on pollen, and here we show adults feeding on open male flowers and also using their rostrum to reach pollen in closed flowers.
